# Magnetic Nanoparticles Interact and Pass an In Vitro Co-Culture Blood-Placenta Barrier Model †

**DOI:** 10.3390/nano8020108

**Published:** 2018-02-14

**Authors:** Elena K. Müller, Christine Gräfe, Frank Wiekhorst, Christian Bergemann, Andreas Weidner, Silvio Dutz, Joachim H. Clement

**Affiliations:** 1 Department Hematology and Oncology, Jena University Hospital, Am Klinikum 1, D-07747 Jena, Germany; Elena.Mueller@med.uni-jena.de (E.K.M.); Christine.Graefe@med.uni-jena.de (C.G.); 2 Physikalisch-Technische Bundesanstalt Berlin, Abbestr. 2-12, D-10587 Berlin, Germany; frank.wiekhorst@ptb.de; 3 Chemicell GmbH, Eresburgstr. 22-23, D-12103 Berlin, Germany; Bergemann@chemicell.com; 4 Institute of Biomedical Engineering and Informatics (BMTI), Technische Universität Ilmenau, Gustav-Kirchhoff-Strasse 2, D-98693 Ilmenau, Germany; Andreas.Weidner@tu-ilmenau.de (A.W.); silvio.dutz@tu-ilmenau.de (S.D.)

**Keywords:** superparamagnetic nanoparticles, BeWo cell line, primary placental-derived pericyte, co-culture

## Abstract

Magnetic nanoparticles are interesting tools for biomedicine. Before application, critical prerequisites have to be fulfilled. An important issue is the contact and interaction with biological barriers such as the blood-placenta barrier. In order to study these processes in detail, suitable in vitro models are needed. For that purpose a blood-placenta barrier model based on the trophoblast-like cell line BeWo and primary placenta-derived pericytes was established. This model was characterized by molecular permeability, transepithelial electrical resistance and cell-cell-contact markers. Superparamagnetic iron oxide nanoparticles (SPIONs) with cationic, anionic or neutral surface charge were applied. The localization of the nanoparticles within the cells was illustrated by histochemistry. The time-dependent passage of the nanoparticles through the BeWo/pericyte barrier was measured by magnetic particle spectroscopy and atomic absorption spectroscopy. Cationically coated SPIONs exhibited the most extensive interaction with the BeWo cells and remained primarily in the BeWo/pericyte cell layer. In contrast, SPIONs with neutral and anionic surface charge were able to pass the cell layer to a higher extent and could be detected beyond the barrier after 24 h. This study showed that the mode of SPION interaction with and passage through the in vitro blood-placenta barrier model depends on the surface charge and the duration of treatment.

## 1. Introduction

Research activities concerning the utilization of nanomaterials in various fields, especially in nanomedicine, have rapidly grown in recent years. Due to the plethora of promising applications of engineered nanoparticles in medicine, the amount of novel nanoparticular formulas is rising, too [1]. Moreover, the occupational exposure to nanomaterials increases more and more, since everyday life products are based on novel nanotechnologies [2]. With respect to the therapeutic application of nanoparticles into the human body, pregnant women in particular embody a specific sensitive group of target persons. Special criteria have to be met when assessing the impact of such materials, especially regarding biodistribution, excretion and biocompatibility. For studying these effects on pregnant women, focus must be especially laid upon the blood-placenta-barrier (BPB), since the placenta is indispensable during pregnancy. Therefore, penetrating or barrier-disrupting nanoparticles might have significant adverse effects onto the fetal development and the course of gestation [3,4]. To appropriately study the effects and potentially associated risks of novel nanomaterials in the placenta and especially the BPB, suitable in vitro models allowing high throughput are most helpful [5].

The BPB, which has the highest interspecies variability among mammals [6], is a highly effective structure responsible for the bidirectional transfer of oxygen, carbon dioxide, water, nutrients, waste products and other substances between the maternal and fetal blood circulations [7]. The actual cellular barrier consists of a continuous layer of the syncytiotrophoblast along with some individual cytotrophoblasts, a thin layer of chorionic connective tissue and the endothelium of the fetal capillary system [3,8]. In between the syncytiotrophoblast layer and the fetal endothelium pericytes are located [9]. During mass transfer, the syncytiotrophoblast layer, which is formed by syncytial fusion of cytotrophoblasts, is the rate-limiting component of the BPB [10]. In the course of pregnancy, the BPB decreases in thickness from over 50 µm in the second month to less than 5 µm at term, due to thinning of the syncytiotrophoblast layer and spreading of cytotrophoblasts, which in turn leads to enhanced transport of substances across the barrier [3,8]. To date, several different models are available to study the behavior and passage of exogenous substances across the human BPB, including in vitro cell culture-based models, ex vivo placental perfusion models or in vivo rodent models [11]. Due to the high species-to-species differences in placental constitution [6], results obtained from animal studies cannot be readily transferred into the context of the human organism. Therefore, models of human origin such as the in vitro transwell BeWo model seem more appropriate. These human choriocarcinoma-derived cells strongly resemble cytotrophoblastic cells, thereby mimicking the precursors of the rate-limiting syncytiotrophoblast layer. Together with their ability to form confluent monolayers on transwell inserts, these cells present a suitable in vitro model to study the transfer of a number of substances, including nanoparticles, through the BPB. BeWo cells have already been used to investigate the transfer of different substances, in particular nanoparticles, through this biological interface [12,13,14].

Magnetic nanoparticles, especially superparamagnetic iron oxide nanoparticles (SPIONs), have remarkable and unique physiochemical properties that discern them from their atom and bulk counterparts [13,15]. Therefore, this group of nanoparticles offers a great potential for nanomedical applications, such as contrast enhancement in magnetic resonance imaging (MRI) scans, cell tracking, tissue engineering, drug delivery or hyperthermal therapy [16,17,18]. In contrast to other metallic nanoparticles such as cobalt, nickel and gold, the essential element iron is naturally occurring in the human body. However, several studies imply some cytotoxic effects to varying degree, whereby the surface coating and charge seem to play pivotal roles [19]. For the further utilization of SPIONs to treat or diagnose various complications during pregnancy, the biocompatibility and the effects of these particles onto the BPB must be investigated in advance. Identifying special attributes of these particles that determine their behavior during their interaction with this biological interface will furthermore help in both predicting the effectiveness of novel formulations and designing tailor-made nanoparticles adapted to the distinct application.

In the present study, we established and optimized an in vitro BPB model for investigating the interaction and the passage of SPIONs through this biological interface. We created a co-culture model using primary placental pericytes in addition to commercially available BeWo cells, and compared this model to the BeWo mono-culture to highlight the positive effect of pericytes onto the model. For the subsequent nanoparticle studies, we focused on the impact of the particle charge onto their uptake into and passage through the BPB by using three equal-sized but differently charged SPION types, coated with neutral starch (D), cationic polyethylenimine (PEI) and anionic carboxymethyldextran (CMX). The effects of SPIONs on the barrier integrity were verified using diverse parameters including the transepithelial electrical resistance (TEER), sodium fluorescein (NaFlu) retention and microscopic investigation of histologic cross sections and immunofluorescently stained cell-cell contacts. Finally, the direct and highly-sensitive detection of SPIONs within the distinct compartments was implemented by means of magnetic particle spectroscopy (MPS) and atomic absorption spectroscopy (AAS).

## 2. Results

### 2.1. Co-Culture of BeWo Cells and Pericytes Improves Barrier Tightness of BPB Model

**Figure 1:**
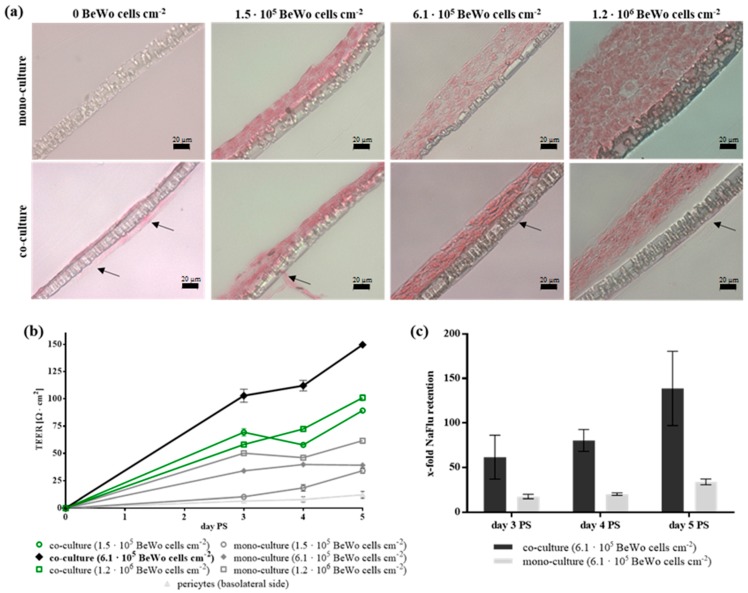
Establishment and verification of an in vitro blood-placenta barrier model. (**a**) Histological cross sections from co-cultures (BeWo, pericytes) and mono-cultures (BeWo) in dependence of the initial cell seeding number. On day five post seeding (PS), histologic cross sections of transwell membranes were stained with Nuclear Fast Red. Arrows mark the pericyte cell layer on the basolateral side of the membrane. Scale bar represents 20 µm. (**b**) The transepithelial electrical resistance (TEER) was measured for each condition in triplicates per insert on days three to five PS and corrected for values of cell-free inserts. Shown are mean TEER values ± standard deviation of three to nine inserts. (**c**) The passage of the permeability marker sodium fluorescein (NaFlu) through the barrier was measured in triplicates for each insert and the calculated permeability coefficients were normalized to blank membranes. For each condition, two replicate inserts were used. Shown are mean values of the x-fold NaFlu retention ± standard deviation of three measurements per insert for two replicate inserts.

In order to study the effect of SPIONs on the blood-placenta barrier, an in vitro co-culture model on the basis of the human cell line BeWo and human primary placental pericytes was established. The setup of the co-culture model is presented in Figure S1. The comparison between the co-culture of BeWo cells and pericytes and the BeWo mono-culture is shown in Figure 1. Histologic cross sections of the transwell membranes revealed the formation of closed apical BeWo cell layers for both the mono- and co-culture models and all varying BeWo seeding densities. Nonetheless, for all seeding conditions, these cell layers appear tighter packed and thinner for the co-culture compared to the mono-culture (Figure 1a). It has to be noted that the detachment of the complete cell layer from the membrane is a technical artefact resulting from the multi-step sample preparation for this analysis method. The barrier formation was further investigated by measuring the TEER and the NaFlu permeability as well as by staining of cell-cell contacts for days three to five post seeding (PS).

Regarding TEER measurements, the values for both models increased with increasing incubation time, while the co-culture model always produced higher TEER values than the mono-culture for all seeding densities (Figure 1b). Thus, on day four PS the co-cultivation of 6.1 × 10^5^ BeWo cells cm^−2^ along with pericytes resulted in the highest value with 112.1 ± 4.8 Ω cm^2^ while the mono-culture of equal numbers of BeWo cells did not exceed 39.8 ± 1.8 Ω cm^2^ and basolateral pericytes alone reached 12.0 ± 3.1 Ω cm^2^.

Observation of the NaFlu permeability showed higher retention of the marker for the co-culture for all investigated days (Figure 1c). The retention values steadily increased for both models, where those of the co-culture reached from 62 ± 25-fold up to a 139 ± 42-fold NaFlu retention relative to cell-free inserts corresponding to permeability coefficients of 5.1 × 10^−7^ ± 2.0 × 10^−7^ cm s^−1^ and 1.5 × 10^−7^ ± 0.5 × 10^−7^ cm s^−1^, respectively. Immunofluorescent staining for the cell-cell contact markers ZO-1 and β-catenin showed the formation of dense BeWo cell layers for both models, while both markers were higher expressed in the co-culture model (Figure S2). Based on these data, confirming the development of a tight in vitro cell barrier using the co-cultivation of 6.1 × 10^5^ BeWo cells cm^−2^ and 1.1 × 10^6^ pericytes cm^−2^ for four to five days PS, these model parameters were used for all further investigations.

### 2.2. Interaction of SPIONs with the BPB Model is Time- and Charge-Dependent without Disrupting the Barrier Integrity

**Figure 2:**
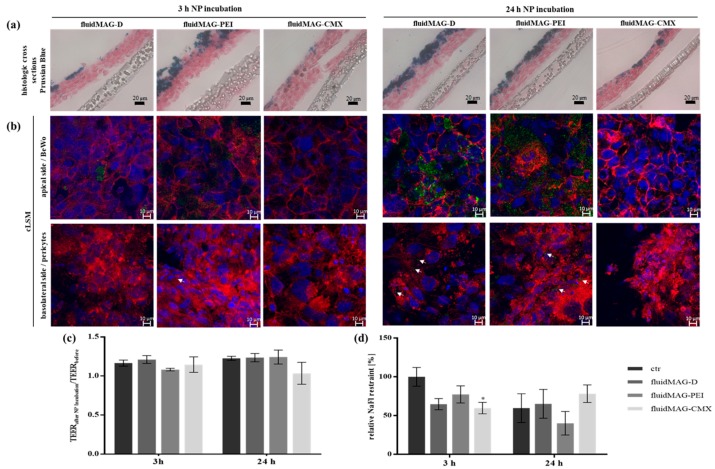
Analysis of the barrier integrity and morphology of the transwell co-culture BPB model after exposure to different SPIONs for 3 h or 24 h. For the co-culture model, 1.1 × 10^6^ cells cm^−2^ of pericytes were seeded onto the basolateral side of 24-well membrane inserts and 6.1 × 10^5^ cells cm^−2^ of BeWo cells were seeded on the apical side of the insert membrane after 24 h. On day four post seeding (PS), barrier models were exposed to 100 µg cm^−2^ (200 µg mL^−1^) of D/PEI/CMX-coated SPIONs for 3 h or 24 h. (**a**) After SPION exposure, histologic cross sections of cell grown transwell membranes were prepared and stained with Nuclear Fast Red and Prussian blue. Scale bars represent 20 µm. (**b**) For the analysis by confocal laser scanning microscopy (cLSM) samples incubated with fluorescently labeled SPIONs (green) were fixed and stained with Hoechst 33258 (blue) and Alexa Fluor^®^ 633 phalloidin (red). White arrows mark SPION aggregates in pericyte cell layer. Scale bars represent 10 µm. (**c**) Transepithelial electrical resistance (TEER) values measured in triplicates per insert before and after SPION exposure were compared for each condition. Shown are the mean values of the quotient of measured TEER values before/after SPION incubation ± standard deviation of three independent experiments. (**d**) The passage of the permeability marker sodium fluorescein (NaFlu) through the barrier after SPION incubation was measured in duplicates for each condition and the calculated permeability coefficients were normalized to blank membranes. Shown are mean values of the x-fold NaFlu retention ± standard deviation of three independent experiments. The significance of the results compared to respective control measurements without SPIONs was tested using two-way analysis of variance (ANOVA) following Tukey’s multiple comparison test. Statistically significant differences are depicted as: * *p* < 0.05. The corresponding *p* values for the comparison of all data sets for (**c**,**d**) are listed in Tables S2 and S3, respectively.

The behavior and interaction of the differently charged SPIONs (Table S1) in the co-culture BPB model were investigated microscopically for exposure times of 3 h and 24 h by means of histologic cross sections and laser scanning microscopy upon fluorescent staining (Figure 2a,b).

Prussian blue as well as fluorescent staining revealed an intensive accumulation of PEI-coated particles in the apical cell layer and intermediate and low accumulation for starch-coated and CMX-coated particles respectively. For starch-coated particles, the accumulation in this layer strongly increased with rising exposure time, in comparison to the other particle formulations. SPION aggregates in the basolateral pericyte cell layer could only be visualized by cLSM for starch-coated particles after exposure for 24 h. In contrast, upon exposure of BPB models with PEI-coated particles, fluorescent particles present within the basolateral pericyte layer can already be detected after 3 h. It has to be noted that in case of PEI-coated particles some cells might appear smaller compared to others. On the one hand that might be a result from disturbing detections of fluorescence signals of phalloidin and/or nuclei due to the severe cellular particle accumulation and associated quenching effects. On the other hand cytotoxic effects of these cationic particles as described earlier [20] and shown in Figure S3 can induce the shrinkage of cells and nuclei. The integrity of the in vitro BPB barrier after SPION exposure was quantified using TEER and NaFlu permeability measurements (Figure 2c,d). The comparison of TEER values after and before SPION exposure revealed no significant alterations of the electrical resistance of the co-culture model, analyzed for all particle formulations and both time points. Regarding molecular permeability, NaFlu retention was slightly but significantly decreased to 59.7 ± 7.3% for models incubated with CMX-coated particles for 3 h compared to the non-treated control after 3 h. Exposure to starch- and PEI-coated particles for 3 h resulted in a non-significant decrease to 64.7 ± 7.2% and 77.3 ± 11.0%, respectively. After 24 h of particle exposure, only the molecular retention of barriers exposed to PEI-coated particles was non-significantly lower than the control with 40.1 ± 15.2%, while starch- and CMX-coated particles even increased the retention capacity of the barriers compared to the 24 h-control (i.e., 59.7 ± 18.6%) to 65.2 ± 18.7% and 78.2 ± 11.3%, respectively.

### 2.3. Passage of SPIONs through the Co-Culture BPB Model Is Subject to the Exposure Time and the Particle’s Surface Charge

**Figure 3:**
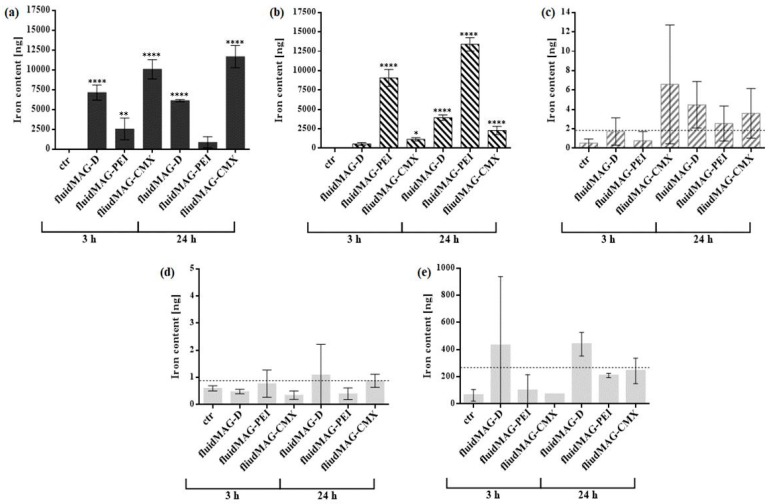
Quantification of SPIONs in the BPB model and investigation of the passage of SPIONs through the barrier. For the co-culture model, 1.1 × 10^6^ cells cm^−2^ of pericytes were seeded onto the basolateral side of 24-well membrane inserts and 6.1 × 10^5^ cells cm^−2^ of BeWo cells were seeded on the apical side of the insert membrane after 24 h. On day four post seeding (PS), barrier models were exposed to 100 µg cm^−2^ (200 µg mL^−1^) of D/PEI/CMX-coated SPIONs for 3 h or 24 h. (**a**–**d**) After SPION exposure, the complete medium of acceptor and donor compartment was collected and cells from apical and basolateral side of the transwell membrane were harvested separately. SPION-associated magnetic iron in each compartment was quantified by magnetic particle spectroscopy (MPS). The magnetic iron amounts within the donor compartment (**a**), the apical BeWo cell layer (**b**), the basolateral pericyte cell layer (**c**) and the acceptor compartment (**d**) are shown as mean values ± standard deviation from two to three independent experiments. For the pericyte layer (**c**) and the acceptor compartment (**d**) the limit of detection (LOD) is depicted as a dotted line. (**e**) After SPION incubation, particles in the acceptor compartment were analyzed for total iron content by atomic absorption spectroscopy (AAS). Shown is the mean ± standard deviation from duplicate measurements. The LOD is depicted as a dotted line. The significance of the results compared to control measurements without SPIONs was tested using two-way analysis of variance (ANOVA) following Tukey’s multiple comparison test. Statistically significant differences are depicted as: * *p* < 0.05, ** *p* < 0.01 and **** *p* < 0.0001. The corresponding *p* values for the comparison of all data sets for (**a**,**b**) are listed in Tables S4 and S5, respectively.

To quantify the passage of the differently charged SPIONs through the BPB model, the total amount of SPION-associated magnetic iron in each of the four transwell compartments was determined by MPS (Figure 3a–d).

Data revealed that PEI-coated particles show the strongest interaction capacity with the BeWo cells, indicated by detected SPION amounts as high as 13.4 ± 0.9 µg after exposure for 24 h, while CMX particles showed the lowest cellular accumulation with 2.3 ± 0.5 µg after 24 h. In concordance with these values, the detected amounts of SPIONs in the upper transwell compartment showed a reverse distribution with 11.7 ± 1.4 µg and 0.9 ± 0.7 µg magnetic iron after a 24-hour incubation with fluidMAG-CMX or fluidMAG-PEI respectively. After a 3 h incubation, only for co-cultures exposed to CMX-coated particles the detected magnetic iron amounts in the pericyte layer were located above the LOD of 1.83 ng, but with profound fluctuations. After 24 h, the values detected for all three SPIONs were located above the LOD. Nanoparticle amounts in the lower compartment were detected in a range below 1 ng of magnetic iron. Since the LOD was appointed to 0.88 ng, very low to undetectable levels of SPIONs were detected to pass the barrier model. Atomic absorption spectroscopy (AAS) was additionally used to quantify the total iron amounts in the lower transwell compartment for the verification of data gained by MPS (Figure 3e). After short-term exposure (3 h), only values for starch-coated particles were observed above the LOD of 191.4 ng within the lower acceptor compartment, although showing profound variations. After 24 h, total iron amounts were observed above the LOD for all three types of SPIONs, but for the neutral starch-coated nanoparticles to the highest extend. For both methods it has to be noted that total iron amounts utilized during the incubation experiments slightly differ between the distinct particle types. The reasons for this are found in the facts that according to both the oxidative state of the superparamagnetic iron oxide core and the relative proportion of surface coating material effective iron concentrations are specific for each particle charge. By keeping the total nanoparticle concentration constant between the different particle formulations diverging effective iron concentrations may come up.

## 3. Discussion

The continuous development of new nanoparticles for various applications, such as in nanomedicine, leads to the increasing need to study the effects of these materials onto the human body. Exposure to nanoparticles during pregnancy has adverse effects for the fetal development, thus studying the impact of nanomaterials onto pregnant women, the placenta, and the fetus is a prerequisite for the application of any nanomedicines during gestation. Prior to biological particle testing in more complex models or in vivo studies, cell culture-based models embody valuable tools for efficient particle pre-screening and high throughput assays.

The BeWo cell line is a well-acquainted and extensively used model for the rate-limiting layer of the BPB. In most studies, the BeWo b30 clone, established by Prof. Schwartz in the 1980s, is used in contrast to the herein used commercial clone [21]. Both cell lines were previously compared concerning the ability to form monolayers and subsequently their feasibility for in vitro transport studies. The b30 clone was found to grow faster and was able to create higher transepithelial resistances on transwell inserts compared to the commercial clone, while both expressed trophoblast-specific markers [12,22]. In this study, the commercial clone was used and extensively characterized with regards to barrier-building properties. In comparison to previous studies, a higher seeding density of 6.1 × 10^5^ BeWo cells cm^−2^ instead of about 1 × 10^5^ cells cm^−2^ for b30 was chosen [12,13,14]. The transepithelial resistances and permeability coefficients measured in this work using the commercial clone for the mono- as well as for the co-culture approach even exceed values of other studies using b30, e.g., by Cartwright et al. (2012) [13]. Histologic cross sections further revealed the formation of a confluent BeWo cell layer as soon as three days post seeding. Nevertheless, no confocal monolayer could be shown, but only multilayers, which indicates that the formation of an intact monolayer is not feasible. In line with this, b30-studies show that obtaining a monolayered BeWo model is challenging due to the lack of growth inhibition upon contact [14]. Therefore, the use of a multilayer model as a reproducible transport model, where the barrier integrity can be assured, seems reasonable [14]. Furthermore, it is noted that the human BPB is composed of multiple layers in early stages of gestation, in particular the syncytiotrophoblast and cytotrophoblasts. Considering all above-mentioned aspects, in this study transport experiments were performed using a BeWo multilayer rather than taking the drawback of sub-confluent monolayers. Additional to BeWo cells, pericytes were used to establish the co-culture model. The communication between the pericytes and the other involved cell types is supposed to be essential for the differentiation of trophoblasts during pregnancy [9]. The optimal conditions were appointed to 6.1 × 10^5^ BeWo cells cm^−2^ on the apical membrane side, 1.1 × 10^6^ pericytes cm^−2^ on the basolateral side and a cultivation time of four to five days PS. The addition of pericytes was shown to increase the transepithelial resistance and the NaFlu retention as well as to improve the morphology of the BeWo cell layer. A synergistic effect of the co-cultivation of BeWo cells together with pericytes could be shown, since TEER values measured for the co-culture were even higher than just adding resistance values for BeWo cells and pericytes alone. Taken these parameters together a barrier of elevated tightness and integrity could be achieved by the mentioned co-cultivation. Further experiments using pericyte-conditioned medium proved that these positive effects of pericytes in the co-culture were not just evoked by the additional cell layer, but also by the direct contact of the two cell types through the membrane pores (data not shown). That indicates the involvement of cell-cell contact-based signaling interactions between these two cell types in addition to secretory factors and cytokines by pericytes. However, more studies need to be performed in order to elucidate the exact underlying mechanism. Furthermore, the investigation of indicated barrier integrity parameters of mono-cultured BeWo cells in presence of pericyte-conditioned medium did not reveal any beneficial barrier-strengthening effects compared to mono-cultured cells exposed to the regular cell culture medium, i.e., DMEM + 10% FCS (data not shown). Thus, a direct physical contact or close proximity of these supporting cells to the BeWo cells seems crucial for the generation of a barrier of advanced tightness. The detailed nature of this direct effect needs to be investigated in more detail by future investigations.

The developed co-culture BPB model was subsequently exposed to the three equal-sized and differently charged SPIONs, coated with neutral starch, cationic PEI or anionic CMX. Investigations of the interaction and uptake of these indicated SPIONs with the co-culture in vitro BPB model revealed the dominant role of the particle charge and exposure time in this context. Cationic PEI-coated particles showed the strongest interaction capacity, while anionic CMX-coated ones showed the lowest susceptibility. In concordance to previous studies [20], cytotoxicity experiments revealed the same tendency, where cationic particles are more toxic to the cells than neutral and anionic ones (Figure S3). The strong interaction and cytotoxicity of cationic particles can be explained by the interaction with the negatively charged cell membrane and the subsequent perforation of the membrane and formation of nanoscale holes, which have been described previously [2,23]. However, the exposure of the BPB model to the differently charged SPIONs for 3 h or 24 h resulted in no significant change of barrier integrity measured by transepithelial electrical resistance. The barrier’s capacity for NaFlu retention increased significantly after exposure for 3 h for CMX-coated particles relative to non-treated controls, while it was not significantly altered for the other conditions and time-points. Only after the exposure of BPB models to PEI-coated nanoparticles for 24 h diminishing but non-significant effects on the barrier’s retention capacity towards NaFlu were observable. Compared to the corresponding nanoparticle-free controls, the NaFlu retention decreased stronger after 3 h than after 24 h, which could be explained by the BeWo cells’ sensitivity to continuous mechanical stress, which is present during NaFlu experiments and might disturb the freshly exposed barrier model. Interestingly, barrier permeability was even decreased for models incubated with D- and CMX-coated particles for 24 h, signaling a barrier-strengthening effect. Furthermore, for the nanoparticle-free control barriers, the permeability increased from the 3 h to the 24 h measurements, indicating that barrier models might be destabilized at about this time span.

The direct quantification of SPIONs in the different compartments of the in vitro model was performed by MPS, a sensitive method to detect the magnetic iron contents in samples and which was already previously established for SPION quantification in an in vitro model of the blood-brain barrier [24]. Results for the upper compartment and the apical BeWo cell layer confirm the previous microscopic interaction studies, which showed that the passage into the cell layers is dependent on the charge of particles and the exposure time. Cationic PEI-coated particles show the most intense interaction capacity dependent on the incubation time, and are therefore incorporated into the BeWo cell layer to the highest extent. In the basolateral pericyte cell layer only small amounts of all tested particles could be detected. Thereby, values hardly exceeded the LOD, indicating the tightness of the co-culture barrier for these SPIONs. With increasing exposure time, there is an incremental tendency to detect more particles in the basolateral cell layer, which hints to a time-dependent transport mechanism of SPIONs through the barrier. These findings are endorsed by the low amounts of SPIONs detected within the lower compartment, which are all located in the range or even below the LOD. It is noted, that before analysis via MPS, the two cell layers of the model had to be detached from the membrane separately and the complete detachment cannot be proved without doubt. To verify the findings acquired by MPS, SPIONs within the lower compartment were also quantified by AAS, which is considered to be less sensitive in comparison to MPS since total iron amounts are quantified [25]. An important difference between MPS and AAS is the way the samples are processed before the actual measurement. MPS samples are passed through many different processing steps which results in higher discrepancies during the measurement, especially for low detectable amounts. Nevertheless, this method was shown to be highly sensitive for the detection of SPIONs in a wide range of 1 ng to many µg [24]. For AAS, on the other hand, sample processing includes fewer steps, but measurement is less sensitive with a narrow detection range. Despite this, the easier sample processing for AAS seems to create more reliable results in this experimental setting, where very small amounts of SPIONs need to be detected. Indeed, values measured by AAS were higher than the ones for MPS, accounting for the different detection techniques. Whereas AAS is based on the detection of total iron, MPS detects (superpara) magnetic—and thus nanoparticle-associated—iron only. Results obtained from the 24 h exposure show same tendencies for MPS and AAS, where starch-coated particles were detected with the highest amounts and PEI-coated ones with the lowest. The comparison of results from 3 h incubations showed larger discrepancies, which suggests that particle passage might strike up in the end of this time span and therefore 3 h represent a critical time point. More detailed analysis will be needed to further quantify the passaged SPIONs. Interestingly, despite especially PEI-coated particles disturbing the cellular barrier, which was shown by NaFlu permeability measurements, lower amounts of these particles were detected in the lower compartment in comparison to the other nanoparticle formulations. A reason for this finding can be found in the agglomeration tendencies of SPIONs in a biological environment, which vary depending on the shape, size and especially the coating and charge of the particles [26,27]. During the conducted experiments, a pronounced agglomeration tendency was found for the cationic PEI-coated particles, which was higher than the ones for the other formulations (data not shown). Since transwell membranes with pore-sizes of 3 µm were used in the passage experiments, large SPION-agglomerations are massively hampered in passing the membrane even when able to pass through the cellular barrier, and can therefore not be detected in the basolateral compartment.

In scope of the present study, a transwell-based in vitro BPB model was established using trophoblast-like BeWo cells and placental-derived pericytes for the further investigation of SPIONs in this biological interface. Our study provided insight into the behavior of SPIONs in the BPB and the influence of the particle charge in this setting. The tight barrier formed by the co-culture was undisrupted by SPION incubation, and was shown to be almost impermeable for the particles. Recently, Blundell et al. introduced a microfluidic-based placenta barrier model, which is based on BeWo cells and human primary placental villous endothelial cells. The microphysiological model is suitable to study transport mechanisms across the barrier under flow conditions as well as during formation of a syncytiotrophoblast recapitulating placental differentiation [28]. Therefore, translation of our static model into a dynamic microfluidic system by incorporation of blood flow conditions is an obvious objective to increase the predictive value of the model. Future studies should include the correlation of these in vitro results to more complex ex vivo or in vivo models like the placental perfusion [12,29]. Concerning the consequences of nanoparticle exposure of pregnant women, while disruption of the BPB by nanoparticles and the direct passage of the particles through the barrier might endanger the fetus, the interaction and passage might be desirable for the direct application of drugs to the fetus or the placenta. The results presented in this study indicate the possibility to selectively target the pregnant mother, the fetus or the placenta by using nanoparticular carriers that effectively pass and interact or are restrained at the BPB. Adding functional groups and ligands to the here used unfunctionalized SPIONs might enhance the selective behavior of the particles to cross or be restrained at the barrier, which can be investigated using the here established in vitro BPB model.

## 4. Materials and Methods

### 4.1. Cell Culture

The human choriocarcinoma cell line BeWo (DSMZ GmbH, Braunschweig, Germany) and primary human placental pericytes (PromoCell GmbH, Heidelberg, Germany) were cultured at 37 °C and 5% CO_2_ in DMEM medium supplied with GlutaMAX-I (Life Technologies, Karlsruhe, Germany), 1% penicillin/streptomycin (10,000 U/mL)(Life Technologies, Karlsruhe, Germany).

For the generation of the in vitro BPB model, 24 well PET transwell membrane inserts with 3 µm pores (Corning, Inc., Corning, NY, USA) were used. One day before seeding of 1.5 ×10^5^–1.2 × 10^6^ cells per cm^2^ of BeWo cells on the apical membrane side, 1.1 × 10^6^ cells/cm^2^ of pericytes were seeded on the basolateral side of the transwell membrane. Cell models were cultivated for three to five days, where culture medium in the upper donor compartment (DC) as well as in the lower acceptor compartment (AC) was renewed every other day. A scheme for the timeline of preparation of the BPB model is shown in Figure S1.

### 4.2. SPIONs and SPION Characterization

All used fluidMAG- and nanoscreenMAG/G-particles were provided by chemicell GmbH, Germany. Spectroscopy was used to quantify the iron concentration of these iron oxide particles using phenanthroline staining. Additionally, the hydrodynamic diameter was measured by dynamic light scattering with the Zetasizer nano series ZS (Malvern Instruments, Herrenberg, Germany). Laser Doppler velocimetry was used to determine the zeta potential of the nanoparticles. All specifications and LOTs from the used SPIONs are listed in the supplement section (Table S1).

### 4.3. Transepithelial Electrical Resistance Measurement

Cell barrier integrity was evaluated by measurements of the transepithelial electrical resistance (TEER) using chopstick electrodes with an epithelial voltohmmeter (WPI, Berlin, Germany). The resistance was measured in triplicates per insert, allied to the effective membrane area (here 0.33 cm^2^) and corrected with background controls (cell-free inserts with respective cell culture medium).

### 4.4. Molecular Permeability Assay

Further verification of the cell barrier’s permeability was performed using sodium fluorescein (NaFlu, 376 Da, Sigma-Aldrich Chemie GmbH, Steinheim, Germany). Transwell inserts were placed in wells containing phenol red-free cell culture medium (=acceptor compartment), while the upper compartment (=donor compartment) was filled with a 2.5 µM sodium fluorescein solution (in phenol red-free medium). The inserts were incubated at 37 °C and 90 rpm with an orbital shaker (30 mm amplitude, GFL GmbH, Burgwedel, Germany) and transferred to new wells containing fresh medium after 10 min. The fluorescence intensity (λex = 460/9 nm, λem = 515/20 nm) of medium samples from the acceptor compartment after indicated time points was measured in triplicate by using the CLARIOstar microplate reader (BMG Labtech GmbH, Ortenberg, Germany). Permeability coefficients *P_NaFlu_* were calculated according to following equation, which was described by Audus and Borchardt [30].

$$P_{NaFlu}=\frac{c_{acc}\;\cdot \;V_{acc}}{t\;\cdot \;A\;\cdot \;c_{do}}$$

*t* = incubation time (s), *A* = effective diffusion area (cm^2^), *c_acc_*/*c_do_* = NaFlu concentration in acceptor/donor compartment (nM), *V_acc_* = volume in acceptor compartment (cm^3^).

### 4.5. Histologic Cross Sections

Thin microtome sections were prepared from transwell membranes to visualize the cell layers. Membranes were cut out of the inserts and embedded in 1% agarose dissolved in PBS after fixation for 15 min with 10% (*w*/*v*) formalin (Sigma-Aldrich Chemie GmbH, Steinheim, Germany). The membrane-containing agarose blocks were fixed again with 10% (*w*/*v*) formalin before dehydration and embedding in paraffin (automatic tissue processor Leica TP1020 (Leica Biosystems Nussloch GmbH, Nussloch, Germany); Leica EG1160 embedding center (Leica Biosystems Nussloch GmbH, Nussloch, Germany). Sections of 15 µm thickness of the membrane-containing blocks were prepared with the Leica RM 2165 automated rotary microtome (Leica Biosystems Nussloch GmbH, Nussloch, Germany). Obtained sections were deparaffinated and hydrated, stained with 2% (*w*/*v*) potassium ferrocyanide (ICN Biomedicals, Aurora, CO, USA) in 1 M HCl for 10 min and counterstained with Nuclear Fast Red (Sigma-Aldrich Chemie GmbH, Steinheim, Germany) for additional 10 min. After dehydration, the sections were embedded with Entellan^®^ New (Merck Millipore, Darmstadt, Germany) and analyzed microscopically.

### 4.6. LSM/Immunofluorescence

For the visualization of SPION distribution within the cellular barrier, cells on transwell inserts incubated with fluorescently labeled nanoparticles (ex/em: 488 nm/588 nm) were fixed with 10% (*w*/*v*) formalin (Sigma-Aldrich, Chemie GmbH, Steinheim, Germany) for 15 min and permeabilized with 0.1% (*v*/*v*) Triton X-100 (Ferak Berlin GmbH, Berlin, Germany) for 10 min. Afterwards, cell nuclei and cytoskeletal structures were counterstained using Hoechst 33258 and Alexa Fluor^®^ 633 phalloidin (both Thermo Fisher Scientific, Schwerte, Germany). Membranes were embedded on microscopy slides using Immu-Mount™ mounting medium (Thermo Fisher Scientific, Waltham, MA, USA and analyzed using the confocal laser scanning microscope LSM 510 META (Carl Zeiss GmbH, Jena, Germany).

### 4.7. SPION Incubation and Quantification via MPS and AAS

After confirmation of cell layer tightness, cell-loaded membrane inserts (co-culture model) were incubated with 100 µg/cm^2^ (200 µg/mL) of neutral fluidMAG-D particles, cationic fluidMAG-PEI (750/O) particles or anionic fluidMAG-CMX, which were added to the upper compartment. The transwell system was incubated on top of a magnet (350 mT at the surface, field gradient 10–15 T m^−1^ at approx. 2 mm) for initial 30 min to accelerate sedimentation of particles, and afterwards incubated for total time span of 3 h or 24 h (Figure S1).

After incubation, the complete upper donor and the lower acceptor compartment were directly sampled. Cell layers from the apical and basolateral side of the transwell membrane were harvested separately by trypsinization. Total SPION-associated magnetic iron amounts within the four compartments were quantified via MPS using a commercial MPS device (Bruker Biospin, Rheinstetten, Germany) operating at a magnetic field B_drive_ = 25 mT and a frequency f_0_ = 25 kHz [31]) as previously described by Gräfe et al. (2016) [32].

In addition, the absolute iron content in the lower compartment was determined by flame AAS with an AAS 5 FL spectrometer (Analytik Jena AG, Jena, Germany). To this end, nanoparticles in the AC were harvested by centrifugation at 20,000× *g* for 30 min before they were dissolved in 32% (*v*/*v*) HCl (Carl Roth GmbH, Karlsruhe, Germany) overnight. After addition of 10% (*w*/*v*) trichloroacetic acid (Carl Roth GmbH, Karlsruhe, Germany and following centrifugation at 3600× *g* for 5 min, the total iron content was detected at a wavelength of 248.3 nm. For the quantification, a calibration curve with defined iron concentrations (0–50 µmol/L) was prepared.

The limit of detection (LOD) for MPS and AAS measurements was calculated for each compartment independently as described in the following equation in accordance to the International Union of Pure and Applied Chemistry (IUPAC) [33].

$$LOD={\bar{X}}_{background}+3\;\cdot \;SD_{background}$$

$\bar{X}$ = mean of background values, SD = standard deviation of background values

### 4.8. Data Analysis

The data obtained from independent experiments with repeated determinations are presented as mean ± standard deviation. For statistical analysis, the software Prism 6 (GraphPad Software, La Jolla, CA, USA) with the two-way analysis of variance (ANOVA) was used. In a following multiple comparison according to Tukey sample means were compared to respective controls. Differences with *p*-values of *p* < 0.05 (*), *p* < 0.01 (**), *p* < 0.001 (***) and *p* < 0.0001 (****) were considered as statistically significant.

## Supplementary Materials

The following are available online at http://www.mdpi.com/2079-4991/8/2/108/s1, Figure S1: Schema for the timeline of preparation of the blood-placenta barrier model Figure S2: Comparison of expression of cell-cell contact markers β-catenin and ZO-1 for mono- and co-culture models, Figure S3: Cellular viability of BeWo cells and pericytes after short-term incubation with SPIONs. Table S1: Detailed characterization and properties of SPIONs, Table S2: Statistical analysis of data shown in Figure 2c, Table S3: Statistical analysis of data shown in Figure 2d, Table S4: Statistical analysis of data shown in Figure 3a, Table S5: Statistical analysis of data shown in Figure 3b.
