# Supplementary material for: Magnetic Nanoparticles Interact and Pass an In Vitro Co-Culture Blood-Placenta Barrier Model
Source: Nanomaterials (Basel). 2018 Feb 14;8(2):108. doi: 10.3390/nano8020108 (PMC5853739; doi:10.3390/nano8020108)
Supplement: Supplementary file 1 [file nanomaterials-08-00108-s001.pdf]

Magnetic nanoparticles interact and pass an *in vitro* co-culture blood-placenta barrier model

Elena K. Müller [1§], Christine Gräfe [1§], Frank Wiekhorst [2], Christian Bergemann [3], Andreas Weidner [4], Silvio Dutz [4] and Joachim H. Clement [1,*]

[1] Department Hematology and Oncology, Jena University Hospital, Am Klinikum 1, D-07747 Jena, Germany
[2] Physikalisch-Technische Bundesanstalt Berlin, Abbestr. 2-12, D-10587 Berlin, Germany
[3] chemicell GmbH, Eresburgstr. 22-23, D-12103 Berlin, Germany
[4] Institute of Biomedical Engineering and Informatics (BMTI), Technische Universität Ilmenau, Gustav-Kirchhoff-Strasse 2, D-98693 Ilmenau, Germany
* Correspondence: joachim.clement@med.uni-jena.de; Tel.: ++49 3641 9325820

Supplementary Materials: The following are available online at www.mdpi.com/link, Figure S1: Schema for the timeline of preparation of the blood-placenta barrier model Figure S2: Comparison of expression of cell-cell contact markers β-catenin and ZO-1 for mono- and co-culture models, Figure S3: Cellular viability of BeWo cells and pericytes after short-term incubation with SPIONs. Table S1: Detailed characterization and properties of SPIONs, Table S2: Statistical analysis of data shown in figure 2(c), Table S3: Statistical analysis of data shown in figure 2(d), Table S4: Statistical analysis of data shown in figure 3(a), Table S5: Statistical analysis of data shown in figure 3(b).

Table S1: Detailed characterization and properties of SPIONs

	**fluidMAG-**		
	-D	-PEI (750/O)	-CMX
LOT	0808/14	2710/14	2006/15
coating	starch	polyethylenimine, MW 750,000 Da	carboxymethyldextran, sodium
core	magnetite	magnetite	maghemite
SPION concentration (solid content)	25 µg/µl	25 µg/µl	25 µg/µl
Fe concentration by phenantroline	10.83 µg/µl	13.24 µg/µl	12.17 µg/µl
hydrodynamic diameter	150 nm	150 nm	150 nm
ζ potential	-11 ± 7 mV	54 ± 12 mV	-24 ± 6 mV

	**nanoscreenMAG/G- (ex/ em: 488 nm/ 588 nm)**		
	-D	-PEI (750/O)	-CMX
sLOT	2406/15	2506/15	1906/15
coating	starch	polyethylenimine, MW 750,000 Da	carboxymethyldextran, sodium
core	maghemite	maghemite	maghemite
SPION concentration (solid content)	25 µg/µl	25 µg/µl	25 µg/µl
Fe concentration by phenantroline	n.d.	n.d.	n.d.
hydrodynamic diameter	150 nm	150 nm	150 nm
ζ potential	-13 ± 9 mV	56 ± 9 mV	-24 ± 6 mV

Table S2: Statistical analysis of data shown in figure 2(c). The significance of the results compared to respective control measurements without SPIONs was tested using two-way analysis of variance (ANOVA) following Tukey's multiple comparison test.

		3h				24h			
		ctr	fluidMAG-D	fluidMAG-PEI	fluidMAG-CMX	ctr	fluidMAG-D	fluidMAG-PEI	fluidMAG-CMX
3h	ctr	/	0.9938	0.8679	0.9999	0.9679	0.9369	0.8995	0.4397
	fluidMAG-D	/	/	0.4584	0.9588	0.9999	0.9999	0.9994	0.1443
	fluidMAG-PEI	/	/	/	0.9600	0.3286	0.2667	0.2216	0.9919
	fluidMAG-CMX	/	/	/	/	0.8851	0.8235	0.7622	0.6152
24h	ctr	/	/	/	/	/	0.9999	0.9999	0.0927
	fluidMAG-D	/	/	/	/	/	/	0.9999	0.0715
	fluidMAG-PEI	/	/	/	/	/	/	/	0.0573
	fluidMAG-CMX	/	/	/	/	/	/	/	/

Table S3: Statistical analysis of data shown in figure 2(d). The significance of the results compared to respective control measurements without SPIONs was tested using two-way analysis of variance (ANOVA) following Tukey's multiple comparison test.

		3h				24h			
		ctr	fluidMAG-D	fluidMAG-PEI	fluidMAG-CMX	ctr	fluidMAG-D	fluidMAG-PEI	fluidMAG-CMX
3h	ctr	/	0.0751	0.4625	0.0317	0.0316	0.0818	0.0010	0.5099
	fluidMAG-D	/	/	0.9331	0.9997	0.9997	0.9999	0.3690	0.9075
	fluidMAG-PEI	/	/	/	0.7343	0.7330	0.9452	0.0543	0.9999
	fluidMAG-CMX	/	/	/	/	0.9999	0.9994	0.6284	0.6868
24h	ctr	/	/	/	/	/	0.9994	0.6298	0.6855
	fluidMAG-D	/	/	/	/	/	/	0.3463	0.9224
	fluidMAG-PEI	/	/	/	/	/	/	/	0.0464
	fluidMAG-CMX	/	/	/	/	/	/	/	/

Table S4: Statistical analysis of data shown in figure 3(a). The significance of the results compared to respective control measurements without SPIONs was tested using two-way analysis of variance (ANOVA) following Tukey's multiple comparison test.

A - upper compartment		3h				24h			
		ctr	fluidMAG-D	fluidMAG-PEI	fluidMAG-CMX	ctr	fluidMAG-D	fluidMAG-PEI	fluidMAG-CMX
3h	ctr	/	< 0.0001	0.0029	< 0.0001	> 0.9999	< 0.0001	0.7491	< 0.0001
	fluidMAG-D	/	/	< 0.0001	0.0031	< 0.0001	0.7700	< 0.0001	< 0.0001
	fluidMAG-PEI	/	/	/	< 0.0001	0.0029	0.0003	0.2060	0.0001
	fluidMAG-CMX	/	/	/	/	< 0.0001	< 0.0001	< 0.0001	0.2564
24h	ctr	/	/	/	/	/	< 0.0001	0.7491	< 0.0001
	fluidMAG-D	/	/	/	/	/	/	< 0.0001	< 0.0001
	fluidMAG-PEI	/	/	/	/	/	/	/	< 0.0001
	fluidMAG-CMX	/	/	/	/	/	/	/	/

Table S5: Statistical analysis of data shown in figure 3(b). The significance of the results compared to respective control measurements without SPIONs was tested using two-way analysis of variance (ANOVA) following Tukey's multiple comparison test.

B - BeWo cell layer		3h				24h			
		ctr	fluidMAG-D	fluidMAG-PEI	fluidMAG-CMX	ctr	fluidMAG-D	fluidMAG-PEI	fluidMAG-CMX
3h	ctr	/	0.6873	< 0.0001	0.0382	> 0.9999	< 0.0001	< 0.0001	< 0.0001
	fluidMAG-D	/	/	< 0.0001	0.7843	0.6873	< 0.0001	< 0.0001	0.0032
	fluidMAG-PEI	/	/	/	< 0.0001	< 0.0001	< 0.0001	< 0.0001	< 0.0001
	fluidMAG-CMX	/	/	/	/	0.0382	< 0.0001	< 0.0001	0.0937
24h	ctr	/	/	/	/	/	< 0.0001	< 0.0001	< 0.0001
	fluidMAG-D	/	/	/	/	/	/	< 0.0001	0.0056
	fluidMAG-PEI	/	/	/	/	/	/	/	< 0.0001
	fluidMAG-CMX	/	/	/	/	/	/	/	/

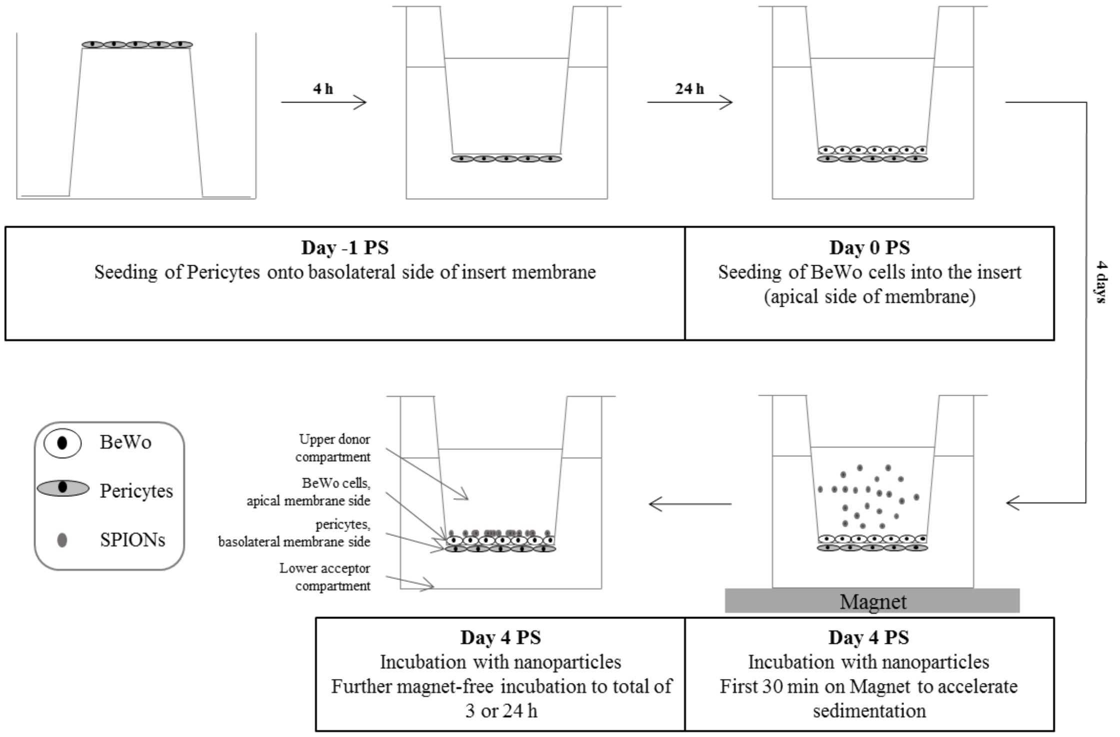

Figure S1: Schema for the timeline of preparation of the blood-placenta barrier model using a co-culture of BeWo cells and pericytes on transwell inserts, including the incubation with SPIONs. PS = post seeding.

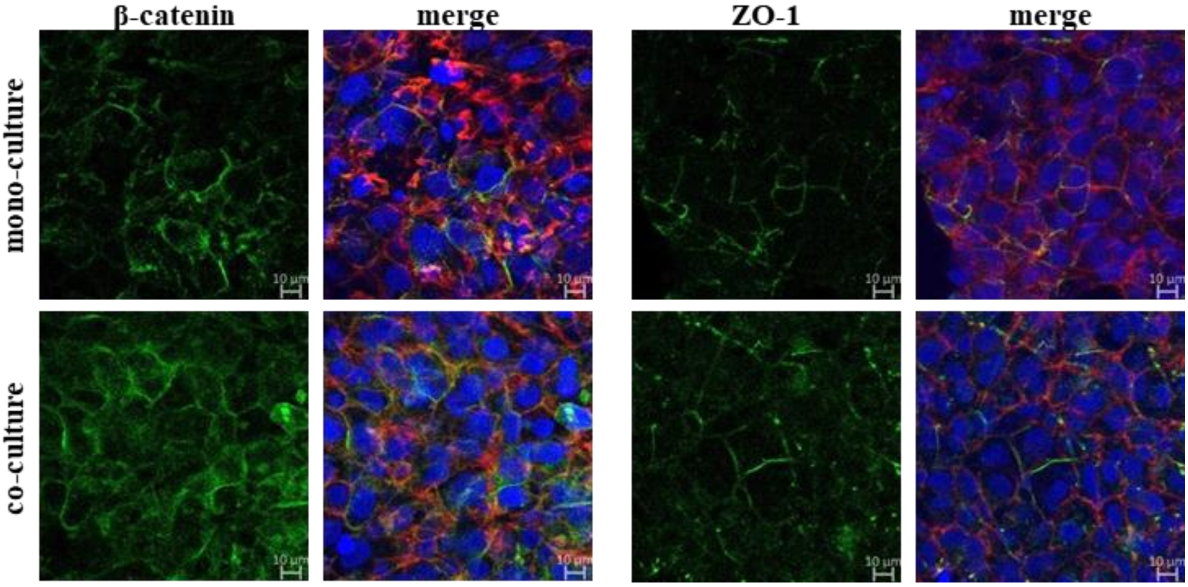

Figure S2: Comparison of expression of cell-cell contact markers β-catenin and ZO-1 for mono- and co-culture models. For the co-culture, $1.1 \cdot 10^6$ pericytes cm^{-2} were seeded onto the basolateral site of 24-well membrane inserts and $6.1 \cdot 10^5$ BeWo cells cm^{-2} were seeded on the apical site of the insert membrane after 24 h. For the mono-culture, only BeWo cells were used. On day four post seeding (PS), cells were fixed, permeabilized and stained with rabbit anti-ZO-1 or β-catenin primary antibody followed by AlexaFluor® 488-labeled goat anti-rabbit secondary antibody (green), Hoechst33258 (blue) and AlexaFluor®633 Phalloidin (red) to visualize cell-cell contacts, cell nuclei and cell cytoskeleton, respectively. Fluorescence signals were acquired by cLSM. Scale bar represents 10 µm.

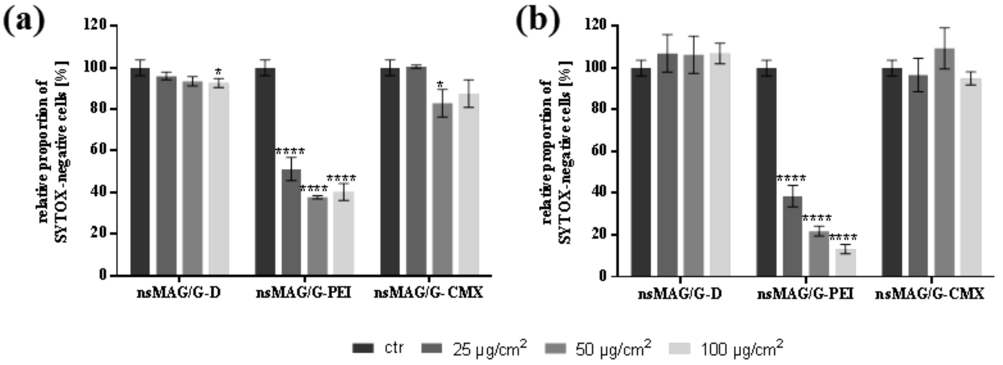

Figure S3: Cellular viability of BeWo cells (a) and pericytes (b) after short-term incubation with SPIONs. 6.6 - 9.2 · 10^4 cells cm^{-2} were seeded into cell culture plates and incubated with 0, 25, 50 or 100 µg cm^{-2} of nsMAG/G-D/PEI/CMX particles for 3 h after overnight cultivation. Afterwards cells were harvested, washed and stained with 2.5 nM SYTOX® red dead cell stain. Analysis of 10,000 events was performed using the FACS Calibur cytometer (BD Biosciences, San Jose, USA) and the obtained data were analyzed using the FlowJo™ software (FlowJo, LLC, Ashland, USA). Shown is the relative proportion of SYTOX-negative cells [%] normalized to diluent-treated cells ± standard deviation for three independent experiments. The significance of the results compared to respective control measurements without SPIONs was tested using two-way analysis of variance (ANOVA) following Tukey's multiple comparison test. Statistically significant differences are depicted as: * $p < 0.05$ and **** $p < 0.0001$.
